# Selection for Replicases in Protocells

**DOI:** 10.1371/journal.pcbi.1003051

**Published:** 2013-05-09

**Authors:** Ginestra Bianconi, Kun Zhao, Irene A. Chen, Martin A. Nowak

**Affiliations:** 1 School of Mathematical Sciences, Queen Mary University of London, London, United Kingdom; 2 Department of Physics, Northeastern University, Boston, Massachusetts, United States of America; 3 Department of Chemistry and Biochemistry, University of California, Santa Barbara, Santa Barbara, California, United States of America; 4 Program for Evolutionary Dynamics, Department of Mathematics, Department of Organismic and Evolutionary Biology, Harvard University, Cambridge, Massachusetts, United States of America; University of British Columbia, Canada

## Abstract

We consider a world of nucleotide sequences and protocells. The sequences have the property of spontaneous self-replication. Some sequences - so-called replicases - have enzymatic activity in the sense of enhancing the replication rate of all (or almost all) sequences. In a well-mixed medium, natural selection would not favor such replicases because their presence equally benefits sequences with or without replicase activity. Here we show that protocells can select for replicases. We assume that sequences replicate within protocells and that protocells undergo spontaneous division. This leads to particular population structures which can augment the abundance of replicases. We explore various assumptions regarding replicase activity and protocell division. We calculate the error threshold that is compatible with selecting for replicases.

## Author Summary

The origin of life, proceeding from chemical reactions to cells, must have included a critical transitional period in which catalytically active sequences arose. A fundamental problem exists for the first catalytic sequences: their activity would not enhance their own fitness directly, and might even decrease their own fitness relative to that of other molecules. Catalytic sequences are constantly encumbered by mutation and drift, limiting the amount of information that can be maintained. Population structures, such as cells, are known to be able to counter this problem. Here we introduce a simple model of the earliest cells to understand limits on information for catalysts with different properties. We find some parallels to information limits on replicators in free solution. Conditions that keep replicases together, or enhance their effect as their abundance increases, permit the evolution of catalytically active sequences.

## Introduction

The origin of life must have required a series of transitions building new levels of molecular interaction. However, a tension often exists between the fitness of an individual sequence and the fitness of the collective [1], [2]. This tension would be important for the earliest replicase enzymes (i.e., replicases), which would help other individuals replicate without helping themselves directly [3]. Indeed, replicase activity cannot be selected in a thoroughly mixed solution, as natural selection favors the evolution of sequences that parasitize the replicases. The proposed solution to this problem is to essentially create small groups of interactors, either by compartmentation or a lattice-like structure [4]–[12]. Selection among individuals in the group favors parasites, but selection at the level of the group favors groups with more replicases, thus allowing ‘altruistic’ replicases to survive [13], [14].

Compartments, in the form of membrane vesicles, have become an important experimental model for protocells [15]–[18]. Amphiphilic molecules, such as fatty acids, that can form membrane boundaries can be produced abiotically [19]–[23] and are found in samples from carbonaceous chondrite meteorites [24]–[27]. Indeed, vesicles can be formed from meteoritic organic extracts dissolved in water [28]. Recent work on model protocell membranes has demonstrated that vesicles can grow through filamentous structures and divide spontaneously by mild shear forces or photochemical stimulation, a robust ‘pearling’ mechanism that produces many small daughter vesicles [29], [30]. Interestingly, experimental studies of cell division mutants in bacteria also suggest that cells divide by pearling when the cell division machinery is eliminated [31]. Pathways for vesicle fission into two daughter vesicles have also been observed, again stimulated by growth [32]–[34]. Ribozyme reactions and non-enzymatic polymerization reactions can be encapsulated inside experimental protocells [35], [36]. Supramolecular assemblies might have a role in promoting polymerization, as demonstrated by the observation that ribozyme-catalyzed RNA polymerization is more efficient if confined to micelles [37]. Inspired by these promising protocell experiments, we focus on vesicles in the theoretical study that follows.

Previous models of compartmentation by vesicles have studied the ability of vesicles to enhance information storage and affect replicase selection. Prior models have usually assumed that the encapsulated genotypes influence vesicle replication (i.e., growth, survival, or division), causing selection among vesicles. Encapsulation has been shown to increase information capacity if vesicle survival depends on the simultaneous presence of multiple self-replicating (i.e., not necessarily replicase) ‘genes’ [4], [10]. In a model by Hogeweg and Takeuchi [7], encapsulation could increase information capacity if the best self-replicators also enhanced vesicle replication, but not if vesicle replication was neutral (i.e., division occurred when the molecular population size reached a certain number). With respect to the evolution of enzymatic activity, replicase dynamics were studied in spatial grids [6], where replicases were found to evolve greater fidelity and information capacity, essentially due to reciprocal altruism in local clusters. However, the generality of this model is unclear, as certain tradeoffs were assumed to exist between replication fidelity, efficiency, and templating ability, and vesicular protocells were not studied. Takeuchi and Hogeweg studied the survival of replicase enzymes (and their parasites) in vesicles, in which parasites were also assumed to contribute to vesicle growth [11]. The dynamics were complicated, but in general selection at the compartment level could counter selection among replicases. Furthermore, stochastic fluctuations have been shown to be important for switching from a ribozyme-poor to a ribozyme-rich regime, a situation that could be enabled by compartmentation into protocells [38], [39].

While recent progress has been made in evolving an RNA enzyme that can copy another RNA sequence [40]–[42], the difficulty of this task has prompted several suggestions for simpler enzymatic activities that might have preceded the polymerase. Any activity that could promote replication would be considered a replicase. For example, RNA sequences that catalyze ligation could stitch together short oligos in a template-directed manner [43]–[46]; an exonuclease could enhance speed and fidelity by removing dangling mismatched ends [47]; a permease could increase the rate of heterotrophic uptake [36]. Cooperative phenotypes may also characterize early autocatalytic replicator cycles, such as systems of ligases or recombinases [43], [48]–[51]. Broadly speaking, in even simpler terms, a replicase might act through colligative properties rather than sequence-specific interactions. For example, an osmolyte might reduce evaporative loss, or a charged polymer might trap useful oppositely charged species. Osmotic pressure has already been shown to drive membrane growth [52]. Such simple chemical activities, while not enzymatic, are weakly altruistic in the sense that they help themselves and other molecules equally.

In light of recent experimental progress, we re-examine the conditions under which enzymatic activity can be selected, using a simple but plausible model of encapsulated replicases and inactive molecules. We first consider a scenario, in which the replicase helps all molecules within a protocell to replicate and is not itself impaired as a template. Then we consider a more altruistic enzyme, which can help other molecules but not itself directly. Vesicle division in our model occurs when the encapsulated population size reaches a certain threshold, but the replicases and vesicles are otherwise unlinked. We calculate the conditions under which altruistic enzymatic activity can be selected.

## Results

We consider two types of sequences. Type 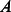 can act as replicase, potentially enhancing the replication of sequences of any type, but type 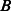 cannot. All sequences undergo spontaneous self-replication, and moreover all sequences are targets of replicase activity. Thus all sequences benefit equally from the presence of type 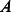 sequences. Type 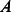 does not have an intrinsic preference to catalyze the replication of other type 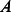 sequences; it treats all targets equally. It is evident that natural selection would not augment the abundance of type 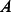 sequences in a well-mixed population. At best, the type 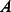 sequences would have the same fitness as all other molecules, so they have no selective advantage. However, 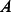 sequences can be erroneously copied to produce 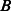 sequences, which causes the population to drift toward an all-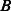 state. Back mutation from 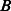 to 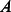 can be neglected, because a specific sequence is needed for replicase activity. Type 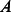 represents a small fraction of possible sequences, while all other sequences are of type 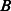.

The probability that a type 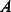 sequence replicates without mutation is given by 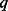. If a type *A* sequence replicates with mutation, the offspring will be a type 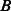 sequence; this happens with probability 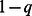. We can think of a point mutation rate, 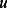, and a number of positions, 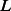, which must remain unchanged in order to retain replicase activity. For example, 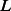 has been estimated to be roughly 75% of the physical length of a functional RNA molecule [53]. Then we have 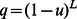. Replication of type 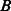 sequences always results in type 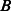; thus we neglect back-mutation. In a well-mixed population type 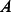 sequences would become extinct for any positive mutation rate, 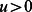.

Let us now study the evolutionary dynamics of 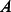 and 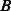 sequences within protocells. Denote by 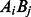 a protocell, which contains 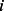 sequences of type 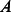 and 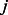 sequences of type 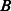. If an 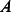 sequence replicates within this protocell without mutation we obtain 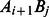. If an 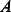 sequence replicates with mutation, or if a 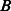 sequence replicates, we obtain 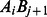.

**Figure 1:**
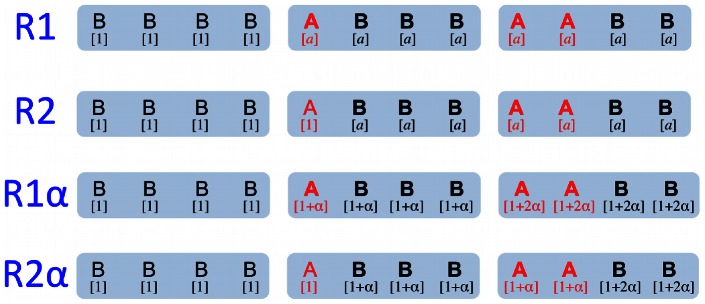
Effect of the replicase in different models. Three possible protocells are shown for each model, each containing four sequences, including 0, 1, or 2 sequences of type *A* (red) and the remainder being type *B* (black). The replication rate of each sequence is shown in brackets under the sequence. Sequences that receive benefit from the replicase(s) are shown in bold type (i.e., replication rate 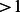). As in the main text, 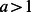 and 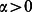. In 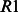 and 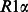, type *A* sequences enhance replication of all *A* and all *B*, such that all sequences in the cell have the same replication rate. In 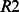 and 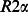, type *A* molecules enhance replication of other molecules but not themselves. In 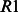 and 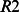, the effect of a single *A* is maximal. In 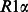 and 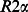, the presence of multiple *A*s increases the rate enhancement.

We explore four different replicases that enhance the replication rate of the molecules within the protocell in different ways. In each case the sequence 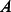 represent a different type of replicase, which we label 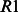, 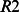, 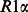 and 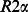 (see Figure 1).

- Replicase 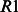 has the following property: the presence of at least one 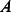 sequence inside a protocell enhances the replication rate of all sequences within that protocell to a value 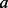, which is greater than 1. In a protocell that contains only type 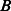 sequences the replication rate is 1.
- Replicase 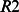 has the following property: the presence of an 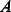 sequence within a protocell augments the replication rates of all other sequences in this protocell, but not its own. Thus, if there is only a single 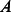 sequence present in a protocell, then all other sequences have an increased replication rate, 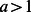, while the 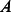 sequence has replication rate 1. If at least two 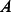 sequences are in a protocell, then all sequences in that protocell have an increased replication rate, 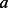.
- Replicase 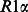 has the following property: the replication rate increases with the number of 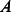 sequences inside a protocell. In particular, we assume that if there are 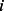 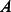 sequences inside a protocell, the replication rate of all sequences within that protocell is 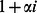, where 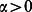. In a protocell that contains only type 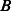 sequences the replication rate is 1.
- Replicase 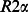 has the following property: the replication rate increases with the number of 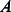 sequences inside a protocell, but a single 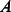 sequence does not enhance its own replication rate. In particular, we assume that if there are 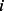 many 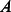 sequences inside a protocell, the replication rate of all *A* sequences within that protocell is 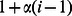, and the replication rate of all *B* sequences within that protocell is 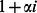, where 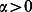. For 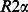, *A* sequences receive less advantage than *B* sequences, as might be expected if the replicase acts directly on other sequences.

**Figure 2:**
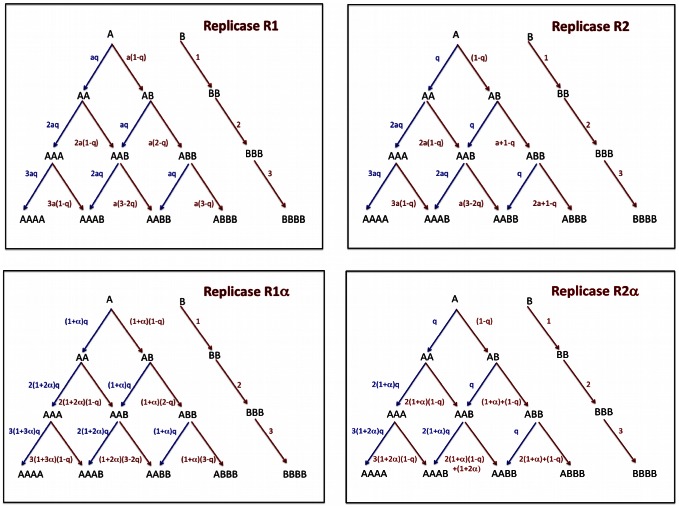
Reaction kinetics for protocells of different composition for the four replicases considered in this paper. The sequences of type *A* indicate the replicases. All sequences undergo spontaneous self-replication and are targets of replicase activity. The sequences of type *B*, in absence of sequences of type *A* in the protocell, replicate at rate 1. A type *A* sequence replicates with mutation with probability 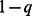, and the offspring will be a type *B* sequence. If the sequences 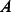 encode the replicase 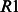, it is sufficient that at least one sequence of type *A* is present in the protocell for enhancing the replication of every sequence in the same protocell to 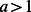. If the sequences 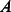 encode for the replicase 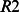, the sequences enhance the rate of replication of all the *other* sequences to 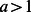. If the sequences 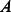 encode the replicase 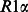, and there are 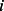 *A* sequences in the protocell, the rate of all the sequences is given by 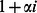 with 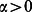. If the sequences 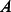 encode the replicase 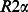, and there are 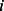 *A* sequences in the protocell, the rate of the *A* sequences is given by 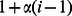 while the rate of replication of the *B* sequences is 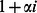 with *α*>0.

In Figure 2 we show the reaction kinetics for all four types of replicases.

**Figure 3:**
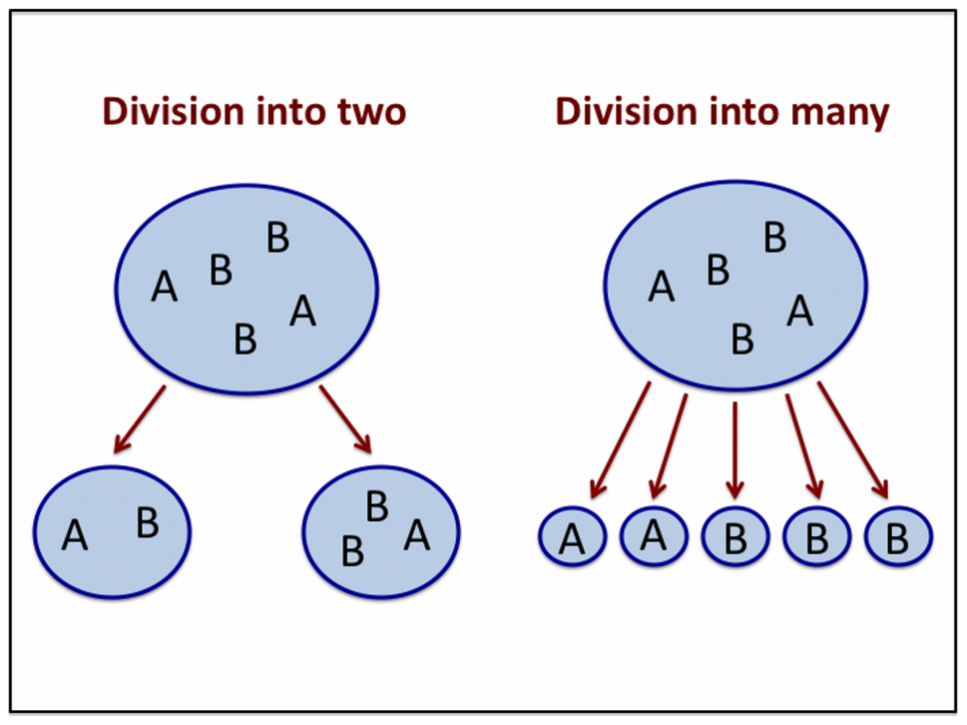
Division mechanism. When a protocell reaches the maximum size 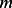, it splits. Here we consider two splitting mechanisms. In the first case the protocell splits into two daughter protocells, of random composition, with each protocell containing at least one sequence. In the second case, the protocell splits into 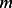 daughter protocells, and each daughter protocell contains a sequence.

Replication within a protocell increases the number of sequences inside the protocell. We assume that the cell divides once a certain maximum number, 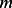, of sequences has been reached. We consider two types of cell division. (i) Division into two: each sequence of the parent cell is given at random to one of the two daughter cells. (ii) Division into many: each daughter cell contains exactly one sequence. In both cases we do not need to keep track of empty cells. In Figure 3 we show how the different mechanisms for cell division work for a protocell of maximal size 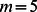.

For replicase 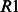 we obtain the following, surprisingly simple result irrespective of the number 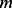 and irrespective of whether cells divide into two or into many. 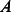 sequences can be maintained in the population if 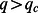 where
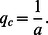
If the probability of error-free replication, 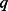, is greater than 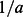, then replicases can be selected within protocells. The result is reminiscent of the error-threshold of quasispecies theory, which describes the selection of a master sequence (not a replicase) in a well-mixed medium [54]–[56].

**Figure 4:**
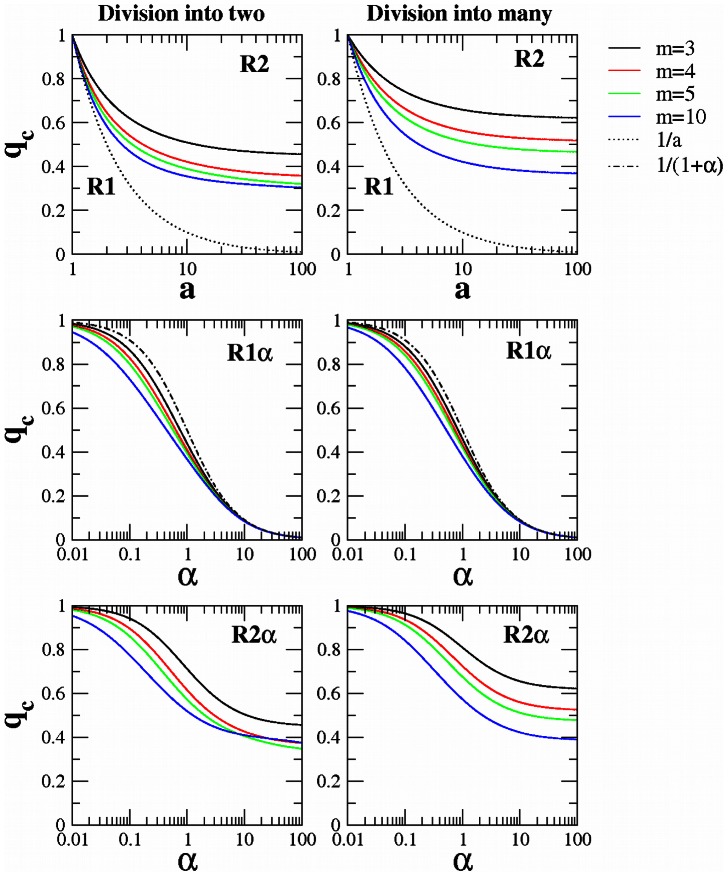
Error threshold for the four replicases under study. In the left panels the protocells of maximal size 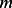 divide into two daughter cells. In the right panels the protocells of maximal size 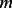 divide into many (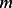) daughter cells. Curves were generated by numerical simulation as described in the text.

For replicase 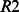 it is harder to select for 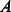 sequences. The reason is that an 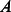 sequence can only help other sequences to reproduce but not itself. Again we find an error threshold, but this time we do not obtain a simple closed form expression. We derive a numerical solution, which is shown in Figure 4. We observe that division into two daughter cells leads to less restrictive conditions (for given 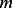) than division into many. In this case if protocells divide into many daughter cells, then each sequence starts off alone within a cell; here single 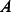 sequences have no advantage over single 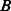 sequences. On the other hand, if protocells divide into two, then for larger 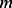 it is typically the case that each 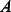 sequences is together with other 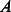 sequences after cell division and immediately benefits from the enzymatic activity.

For replicase 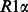, we can prove (see Materials and Methods and Text S1) that the error threshold satisfies the following inequality
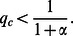
Our numerical solution of the error threshold shown in Figure 4 demonstrates that this upper limit is tight for large values of 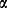. These results are valid when we consider division into two as well as division into many.

Replicase 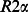 can only help other sequences to reproduce but not themselves. Therefore we find that 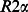 is more difficult to select than 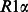 (see Figure 4). We observe that division into two leads to less restrictive conditions (for given 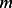) than division into many. We can explain this phenomenon with the same argument used to explain why it is more difficult to select for 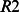 than for 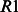 (see above).

We have conducted numerical calculations of the error threshold for values of 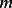 between 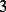 and 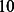. We observe that for 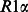 the error threshold is a decreasing function of the maximum number of encapsulated sequences 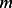. We find a similar behavior of the error threshold for 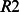 and 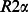 as long as the protocells split into many daughter cells. In this case, for larger 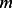 it is easier to select for these replicases. But if the protocells divide into two, we observe for 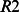 and 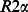 that the error threshold for a given 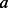 (or 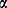) does not always decrease with 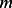. Instead there is an optimum cell size which favors selection of the replicase.

## Discussion

We have studied the constraints on the information content of a replicase arising in protocells. Mutations that produce inactive variants of the replicase are an inevitable consequence of molecular replication, creating parasitic or commensal sequences that derive benefit from the presence of the replicase. The population structure imposed by the protocell membranes can prevent loss of the replicase. We investigated four types of replicases and two types of division. Table 1 summarizes our results, showing the maximum genome length 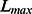 (i.e., number of invariant, informative sites), given experimental parameters for error rates and a prebiotically plausible replication enhancement from the presence of the replicase. The experimental error rates cover a reasonable range, from the misincorporation rate of non-enzymatic RNA replication (representing the lowest fidelity reaction one might consider) to the error rate of an RNA polymerase replicase producing full-length copies (a recently reported replicase with relatively high fidelity) [40], [57]. The results also depend on the value of 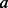 or 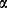. In Table 1, a low value was chosen (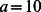, 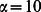) to represent early, relatively poor replicases, but the absolute numbers would increase with greater replicase activity (See Table S1 in Text S1 for analogous calculations for 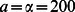 ). It is also important to note that 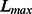 is the number of invariant sites, so the physical length of the molecule could be greater [53]. In addition, very small replicases have been reported (as small as 5 nt; [58]), so it is conceivable that low-information sequences could potentially act as weak replicases.

**Table 1:** Maximal length of the selected replicase 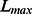.

Division into two	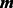	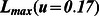	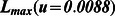
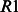	any 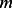	12	260
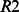	3	3	76
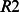	4	4	97
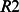	5	5	106
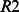	10	5	117
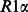	3	13	275
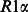	4	13	277
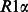	5	13	278
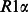	10	13	280
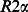	3	3	77
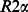	4	4	96
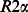	5	4	102
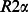	10	4	100

Maximal length of the selected replicase 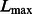 calculated by imposing 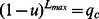 for the different models under consideration with 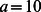 or 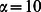. The parameter 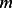 is the maximum number of sequences in the protocell. The parameter 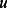 is the mutation rate per base. The parameters 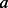 and 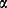 reflect the rate enhancement from type *A* sequences. 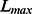 may be roughly 75% of the physical length of the molecule for functional RNA [53].

There are many possible chemical functions that could enhance molecular replication within the cell. Two major categories of replicases are those whose presence helps all molecules in the cell, including itself (a commensal situation; replicases 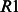 and 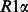), and those whose presence helps other molecules in the cell but not itself (an altruistic situation; replicases 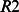 and 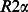). Commensal ‘replicases’ might have beneficial colligative properties. These represent a very early stage of evolution, in which sequences did not necessarily perform specific functions and could be poorly folded. For example, this situation might apply to the selection of the chemical backbone (e. g., RNA) itself. On the other hand, altruistic replicases might perform any number of specific functions, and indeed any RNA that folded into a stable structure would have a compromised fitness for template replication compared to poorly folded RNAs. Intuitively, it is therefore more difficult for an altruistic replicase to survive, so less information can be maintained, as we observe in our results (Table 1).

One analytical result of particular interest is the form of the error threshold for 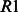 in protocells (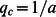). This form is identical to the form of the classical error threshold considering a ‘master’ replicator sequence with fitness 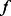 competing with its mutants (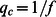) [59], [60]. If enzymes are encapsulated in protocells, it seems that selection has effectively moved up to the next level, from competition among individual replicator sequences to competition among protocells based on the encapsulated genotypes. The collective advantage 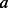 takes the place of the individual advantage 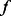, and the survival of the enzyme depends on the mutation rate just like a ‘master’ sequence would in free solution.

The dependence of replication enhancement on the number of replicases is likely to increase linearly initially, and then to saturate at some point. We examined these two regimes separately. To examine the saturated regime, we assumed that a single copy of 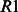 (commensal) or 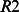 (altruistic) produced the maximal effect on replication rates. 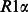 (commensal) and 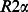 (altruistic) represent the analogous initial regimes, respectively. These two regimes give similar limits on information, particularly at large values of 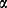, but slightly more information could be maintained in the initial, non-saturated regime. Intuitively, if protocells containing multiple replicases have greater advantage, the overall benefit from the presence of the replicase is greater, allowing more information to be maintained at the same mutation rate.

Cell division typically proceeds via binary fission, or division into two daughter cells. In addition, some model protocells divide by fissioning into many daughter vesicles as described earlier. Bacteria lacking cell division machinery also appear to divide by fissioning into many small cells [31]. We therefore modeled two limiting scenarios for division mechanisms: division into two daughter vesicles, and division into many daughter vesicles (i.e., more daughter vesicles than encapsulated replicators, Figure 3). In general, binary fission is better in terms of maintaining genetic information. Intuitively, binary fission can keep replicases together so they benefit from one another, while division into many vesicles immediately isolates the replicases from each other.

Like previous theoretical models [7], we assume that division occurs upon reaching a particular size 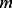 (a number of encapsulated sequences). In general, larger 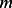 is more permissive to the replicases, allowing more information to be stored because replicases can group together more of the time, enhancing the mutual benefit. In our case, for R1, there is no disadvantage for isolated single replicase molecules, and we obtain that group size is irrelevant to the error threshold. For R2, the replicase is disadvantaged compared to the mutant sequences, but the addition of mutant sequences does not further decrease the fitness of the replicase; in addition, if two replicases are present, then the replicases do not suffer the disadvantage in the protocell.

The effect of cell size, 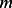, is in contrast with group selection models based on cooperator-defector games, in which larger group size makes selection of the cooperator trait more difficult [9]. In those models, larger groups are more likely to generate defectors (by mutation), which then take over the entire group because of their intrinsic selective advantage. In our current model, larger cells are also more likely to produce non-catalytic (type 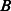) sequences by mutation, but they do not have a selective advantage; they rely on mutation pressure and drift to take over a cell.

To summarize, an RNA replicase arising during the origin of life would be most able to resist mutational pressure under the following conditions: the ability to enhance its own fitness, compartmentalization (which permits selection of the enzymatic behavior), additive enhancement from multiple replicases, larger cell size, and binary fission of compartments. The replicases might correspond to a number of different possible chemical activities. For example, 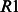 and 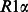 could correspond to a bulk chemical activity (e.g., charged polymer) that enhances replication for all encapsulated sequences (e.g., by attracting oppositely charged ‘food’ molecules) without impacting its own replication. 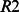 and 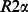 could correspond to a ribozyme with a specific folded structure, which benefits other sequences but not itself directly, such as an RNA polymerase or a membrane transporter.

In conclusion, we have attempted to present the simplest possible models for the selection of enzymatic activity that are inspired by experimental protocells. We estimate the conditions that enable survival of the replicase trait. We focused on simple models in order to understand the underlying dynamics. However, this work could potentially be extended to include more realistic chemical detail, as found in other recent modeling [61]. Other processes could also be included, such as exchange of genetic material among protocells [62]. Another important consideration is that our modeling is deterministic, as a first step in understanding the system. Although the number of RNAs per protocell is small, the number of protocells may be large, justifying a deterministic approach. However, a stochastic approach would be more realistic and could highlight interesting phenomena [38], [39]. Also, in our model, we assume that some *A* is present in the initial pool, and therefore survival of *A* depends on the error criterion. Because we neglect back-mutation of *B* into *A*, *A* cannot be generated de novo in our model; a more realistic model would include the possibility of back-mutation. In addition, our model includes the decay or degradation of protocells (and thus the replicases contained within them), but not of individual sequences within the protocells. This corresponds to the assumption that the removal of protocells (e. g., by dilution), rather than destruction of individual sequences, is the dominant process of decay. Thus, a system containing *A*'s could transition to an all-*B* system through loss of protocells containing *A*'s. A more realistic model would include differential decay of molecules within the protocells as well. Further studies would be needed to test the effects of such realistic modifications to the models. Notwithstanding additional complexities, we find that replicases can be selected under a variety of assumptions. In the simplest case we observe an error threshold arising from protocell competition, in striking analogy to replicator competition. That is, the condition for replicase selection in protocells mirrors the classical condition for replicator selection [54], [59], suggesting the emergence of a new level of selection in which protocells are a mathematical analog to replicators. In addition, we find that conditions that tend to keep replicases together, or enhance their effect as their abundance increases, permit evolution of more information.

## Materials and Methods

### The mutation-selection-cell division (MSCD) equations

We indicate with 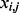 the frequency of protocells of composition 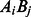. In the Text S1 we describe the mutation-selection-cell division (MSCD) equations for the general case. Here we show how the model reads for replicase 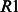. The reaction kinetics are described in Figure 2. The MSCD equations for replicase 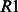 read
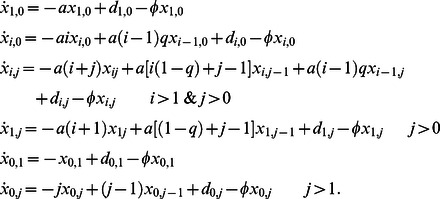
In these equations 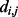 denotes the rate at which protocells of composition 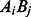 are formed as a consequence of the splitting of protocells of size 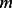. For splitting into two daughter cells, 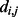 can be written as
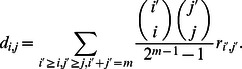
For splitting into many (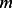) daughter cells, 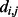 can be written as
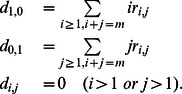
where the dissociation rates 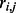 of protocells with 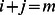 are given by
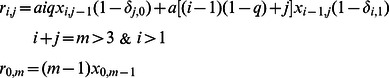



### The error threshold for replicase 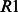


The frequency of sequences A (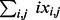) evolves according to the MSCD equations and can be written as
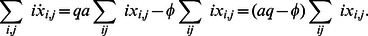
Therefore if
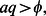
the number of protocells with sequences A increases. On the other side the total number of sequences 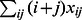 evolves according to the MSCD equation and it can be proved that independently of the splitting mechanism, it satisfies the following equation:
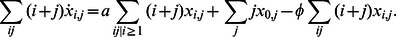
By setting 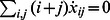 we obtain the value for 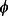 that is needed to preserve the total number of sequences ( i.e. 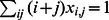). We find therefore
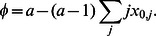
Therefore 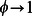 if 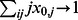. Substituting 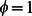 in the relation 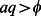, we find that the configuration with 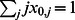 is not stable and protocells with sequences A will be selected if
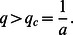
This result is proved here for the case in which we assume that the number of sequences in the system remains constant. Nevertheless the error threshold of the model remains the same if we impose that the number of protocells in the system is fixed. In the Text S1 we give full details of this derivation and we show how to solve the MSCD equations for the other replicases considered in this paper.

## Supporting Information

Text S1 **Detailed description of model and calculations.**
(PDF)
