# Supplementary material for: Selection for Replicases in Protocells
Source: PLoS Comput Biol. 2013 May 9;9(5):e1003051. doi: 10.1371/journal.pcbi.1003051 (PMC3649988; doi:10.1371/journal.pcbi.1003051)
Supplement: Text S1 — Detailed description of model and calculations. (PDF) [file pcbi.1003051.s001.pdf]

Selection for replicases in protocells: Text S1

Ginestra Bianconi, Kun Zhao, Irene A. Chen & Martin A. Nowak

I. THE GENERAL FRAMEWORK

We consider protocells containing self-replicating sequences of type A and type B. Self-replicating sequences of type A act as replicases and are able to speed up the replication of other sequences A and B in the same protocell. The rate of replication of sequences B in absence of sequences A is assumed to be a constant that we set to 1. The probability that a sequence A replicates without mutation is given by $q = (1-u)^L$ where u is the probability of point mutations and L is the length of the sequence.

A. Transition rates

We distinguish between five types of replicases:

- **Replicase R1.**
  If there is at least one sequence A (replicase $R1$) in the protocell, the replication of all the sequences occurs at rate $a > 1$.

- **Replicase R2.**
  If there are two or more sequences A (replicases $R2$) in the protocell, the replication of all the sequences occurs at rate $a > 1$. If only one sequence A is present in the protocell, this sequence replicates at rate 1, while the B sequences in the protocell replicate at rate a.

- **Replicase R1α.**
  If there are i sequences A (replicases $R1\alpha$) in the protocell, the replication of all the sequences occurs at rate $1 + i\alpha$ with $\alpha > 0$.

- **Replicase R2α.**
  If there are i sequences A (replicases $R2\alpha$) in the protocell, the replication of all the A sequences occurs at rate $1 + (i-1)\alpha$ and the replication of the B sequences occurs at rate $1 + \alpha i$ with $\alpha > 0$.

B. Division mechanisms

The division of the protocells can occur according to different dynamical rules. We have considered the following two division mechanisms:

- **i.** The protocell, when it reaches a certain cell size m, splits into two smaller protocells each one containing at least one sequence.

- **ii.** The protocell, when it reaches a certain size m, splits into m protocells each one containing a single sequence.

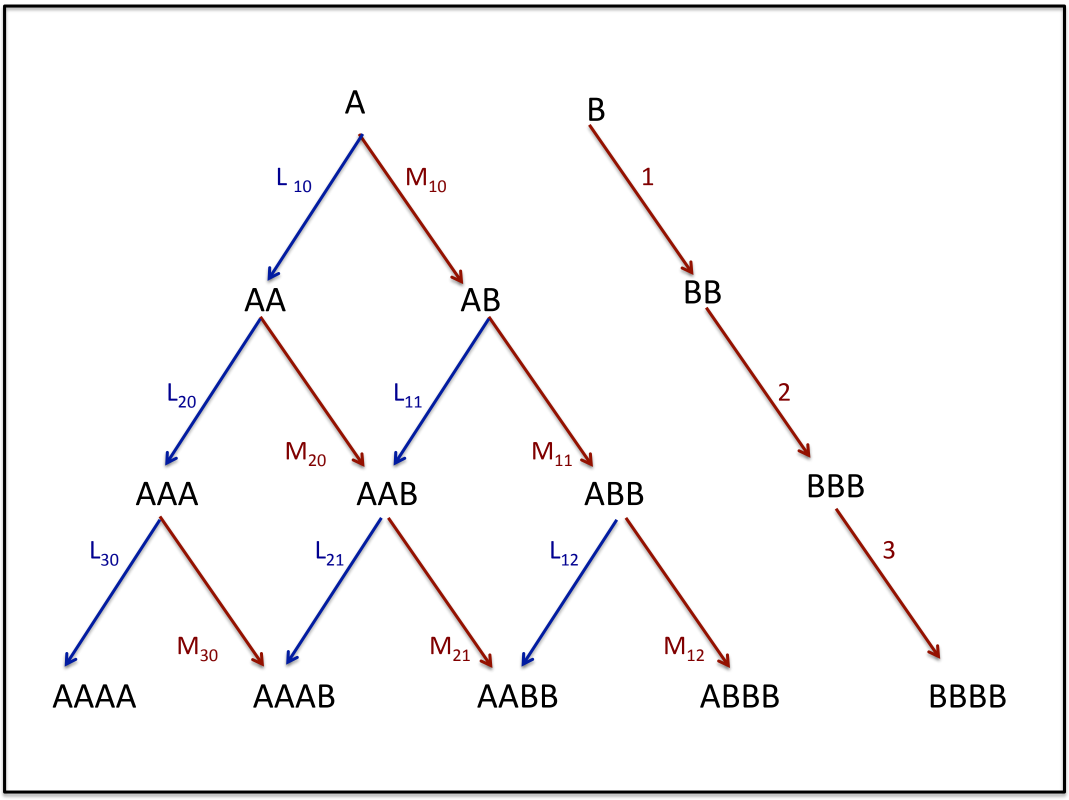

FIGURE S1 : General reaction kinetics for the evolution of protocells. The protocell composition includes both sequences of type A (replicases) and sequences of type B. Both sequences can self-replicate but sequences of type A (replicases) are able to speed up the replication rate of the other molecules inside the same protocell. When a sequence of type A replicates, a mutation occur with probability $1 - q$, giving rise to a sequence of type B. When a protocell reaches a maximum size, it splits.

II. GENERAL RESULTS

A. The mutation-selection-cell division (MSCD) equations

We indicate with $x_{i,j}$ the frequency of protocells of composition A_iB_j. The reaction kinetics for a general model of evolution of protocells is described in Figure S1. The MSCD equations read in the general case

$$\begin{aligned}
\dot{x}_{1,0} &= -[M_{1,0} + L_{1,0}]x_{1,0} + d_{1,0} - \phi x_{1,0} \\
\dot{x}_{i,0} &= -[M_{i0} + L_{i0}]x_{i0} + L_{i-1,0}x_{i-1,0} + d_{i,0} - \phi x_{i,0} \\
\dot{x}_{i,j} &= -[M_{ij} + L_{ij}]x_{ij} + M_{i,j-1}x_{i,j-1} + L_{i-1,j}x_{i-1,j} + d_{i,j} - \phi x_{i,j} \qquad i > 1 \& j > 0 \\
\dot{x}_{1,j} &= -[M_{1j} + L_{1j}]x_{1j} + M_{1,j-1}x_{1,j-1} + d_{1,j} - \phi x_{1,j} \qquad j > 0 \\
\dot{x}_{0,1} &= -x_{0,1} + d_{0,1} - \phi x_{0,1} \\
\dot{x}_{0,j} &= -jx_{0,j} + (j-1)x_{0,j-1} + d_{0,j} - \phi x_{0,j} \qquad j > 1.
\end{aligned} \tag{1}$$

In these equations the rates $M_{i,j}, L_{i,j}$ depend on the particular model under consideration and they will be specified for any particular model taken in consideration in the following sections. Here we focus on the general aspects of the MSCD equations which are independent of the model specification. In Eqs. (1) $d_{i,j}$ denotes the rate at which protocells of composition A_iB_j are formed as a consequence of the division of protocells of size m. For division into two daughter cells, $d_{i,j}$ can be written as

$$d_{i,j} = \sum_{i'\geq i, j'\geq j, i'+j'=m} \frac{\binom{i'}{i}\binom{j'}{j}}{2^{m-1}-1} r_{i',j'}. \tag{2}$$

For division into many (m) daughter cells, $d_{i,j}$ can be written as

$$\begin{aligned} d_{1,0} &= \sum_{i\geq 1, i+j=m} i r_{i,j} \\ d_{0,1} &= \sum_{j\geq 1, i+j=m} j r_{i,j} \\ d_{i,j} &= 0 \qquad (i>1 \; or \; j>1). \end{aligned} \tag{3}$$

In Eq. (2) and Eqs. (3) the dissociation rates $r_{i,j}$ of protocells with $i+j=m$ are given by

$$\begin{aligned} r_{i,j} &= L_{i,j-1}x_{i,j-1}(1-\delta_{j,0}) + M_{i-1,j}x_{i-1,j}(1-\delta_{i,1}) \quad i+j=m>3\&i>1 \\ r_{0,m} &= (m-1)x_{0,m-1} \end{aligned} \tag{4}$$

The parameter ϕ is the fitness of the population of protocell, and we can use that to normalize either the total number of protocell in the system $\sum_{ij} x_{ij} = 1$ or the total number of molecules $\sum_{ij}(i+j)x_{ij}$. The results on the error threshold are independent on the type of normalization adopted.

B. The error threshold: implicit equation

In order to find an equation for the error threshold we investigate the structure of the MSCD Eqs. (1). We define a vector $\vec{y}^A$ including the frequency of protocells containing at least one sequence A,

$$\vec{y}^A = \{x_{i,j}\}_{i\geq 1}. \tag{5}$$

Similarly we define a vector $\vec{y}^B$ including frequency of protocells containing only sequences B,

$$\vec{y}^B = \{x_{0,j}\}_{j\geq 1}. \tag{6}$$

The quasi-species equations given by Eqs.(1) can be written as

$$\begin{aligned} \dot{\vec{y}}^A &= C_{AA}\vec{y}^A - \phi\vec{y}_A \\ \dot{\vec{y}}^B &= C_{BA}\vec{y}^A + C_{BB}\vec{y}^B - \phi\vec{y}^B \end{aligned} \tag{7}$$

where C_{AA}, C_{BA}, C_{BB} are matrices whose explicit expression can be found by considering Eqs.(1). The stationary solution is reached when $\phi = \lambda_{max}$ where λ_{max} is the maximal eigenvalue of the eigenvalue problem

$$C_{AA}\vec{y}^A = \lambda\vec{y}^A$$

$$C_{BA}\vec{y}^A + C_{BB}\vec{y}^B \;=\; \lambda\vec{y}^B. \tag{8}$$

To calculate the error threshold $q_c(a)$, in the appendix A we show that one solution exist with $\vec{y}^A = \vec{0}, \vec{y}_i^B > 0 \forall i$ exists and the maximal eigenvalue associated with such solution is $\lambda_{max} = 1$. In the appendix we will also show that this result is independent on the type of splitting mechanism adopted.

Therefore the error threshold of the models are provided by the following implicit equation

$$|C_{AA} - \phi I| = |C_{AA} - I| = 0. \tag{9}$$

We have used this equation to find the error threshold for all the models under consideration. In the case in which this equation has multiple solutions $q = q_c \in (0, 1]$, according to the MSCD equation, we have to find the solution $q = q_c$ for which $\phi = 1$ is the maximal eigenvalue of the matrix C_{AA}. In order to give a concrete example on how this calculation is performed in appendix B we give the analytic solution of the error threshold for the replicase R2 with $m = 3$.

C. The error threshold: General relation

Let consider the problem of finding a non zero solution $\vec{y}^A \neq \vec{0}$ to the problem

$$C_{AA}\vec{y}^A = \vec{y}^A \tag{10}$$

as long as $q = q_c(a)$. The system of equations that we need to solve is then

$$x_{i,j} \;=\; -[M_{i,j} + L_{i,j}]x_{i,j} + M_{i,j-1}x_{i,j-1}(1 - \delta_{j,0}) + M_{i-1,j}x_{i-1,j}(1 - \delta_{i,1}) + d_{i,j} \qquad i \geq 1 \tag{11}$$

Multiplying each of these equation by i and summing over i and j we can prove that at $q = q_c(a)$ the frequencies of protocells $x_{i,j}$ should satisfy

$$\sum_{i\geq 1,j} ix_{i,j} = \sum_{i\geq 1,j} L_{i,j}x_{i,j}. \tag{12}$$

In fact it can be shown that

$$\sum_{i,j} id_{i,j} = \sum_{i,j,i+j=m} ir_{i,j} \tag{13}$$

independently from the splitting method taken under consideration. Eq. (12) is a very useful relation to determine some bounds for the error-threshold of the model. Nevertheless this equation doesn't solve the entire MSCD model because it depends on the values of the frequency of the protocells $x_{i,j}$.

III. SPECIFIC MODELS

A. Replicase R1

In this model if there is at least one sequence A in the protocell, the replication of all the sequences occur at rate $a > 1$. We consider at the same time the two proposed mechanisms for protocell division.

The rates $L_{i,j}, M_{i,j}$ are given by

$$\begin{aligned} L_{i,j} &= iaq \\ M_{i,j} &= ia(1-q) + ja. \end{aligned} \tag{14}$$

Considering the general relation Eq. (12) and substituting the rates given by Eqs. ($B12$) we get that, at the error threshold, the frequencies $x_{i,j}$ satisfy

$$\sum_{i\geq 1,j} ix_{i,j} = aq_c \sum_{i\geq 1j} ix_{i,j} \tag{15}$$

Therefore

$$q_c = \frac{1}{a} \tag{16}$$

independently of the maximal size of the protocell m and the division mechanism.

B. Replicase R2

In this model the enzyme A is able to speed up the reaction of other molecules (either sequences A or B) but not itself. Therefore the rates of duplications are given by

$$\begin{array}{lcll} L_{i,j} &=& q & \text{for } \; i=1 \\ L_{i,j} &=& iaq & \text{for } \; i\geq 2 \\ M_{i,j} &=& aj+(1-q) & \text{for } \; i=1 \\ M_{i,j} &=& aj+ai(1-q) & \text{for } \; i\geq 2 \end{array}$$

By using the relation Eq. (12) valid at the error threshold, we get that

$$\sum_{i\geq 1,j} ix_{i,j} = aq_c \sum_{i\geq 1} ix_{i,j} - q_c(a-1)\sum_j x_{1,j} \tag{17}$$

Since we assume $a > 1$ we can show that with these rates the selection of an enzyme is more difficult than in the previous model and we have

$$q_c \geq \frac{1}{a}. \tag{18}$$

Moreover by solving the Eqs. (1) in the large a limit we can show that in this limit

$$\begin{array}{lll} x_{1,0} &= d_{1,0}/2 = \mathcal{O}(1) & \\ x_{i,j} &= \mathcal{O}\left(\frac{1}{a}\right) & \text{for } \; i\geq 1 \& (i,j)\neq(1,0) \end{array}$$

Therefore, taking into account this scaling and Eq. (17) we can show that

$$q_c(a) \to q_c(\infty) > 0 \;\; \text{as} \;\; a\to\infty \tag{19}$$

We have numerically calculated the error threshold for this model up to $m = 10$ considering both types of protocell division, i.e. the case in which each protocell of maximal size m splits into two protocells or the case in which it splits into many protocells. The results show, as the theory and Eq. (19) predict, that in this case the selection for

sequences of type A (replicases $R2$) is more difficult than the selection of replicases $R1$ considered in the previous section.

The simulation results conducted for the case in which the protocells of maximal size divide into two, show a crossing of the error threshold curves $q_c = q(a)$ calculated for different values of the maximal size of the protocell m. For example, we observe that, for $a = 200$, the error threshold for $m = 8$ is smaller than the error threshold for $m = 10$ (See Table S1), while for $a = 10$, we observe that the error threshold for $m = 8$ is greater than the error threshold for $m = 10$. This suggests that, for a value of $a = 200$, there is an optimum size of the protocell. We have performed the numerical calculation up to value of $a = 10,000$ for protocells of maximal size $m \in [3, 10]$. These results suggest that in the large a limit ($a \gg 1$) the error threshold q_c is a decreasing function of m.

The simulation results conducted for the case in which the protocells of maximal size divide into many, show that, for every value of a the error threshold is a decreasing function of m. Therefore if the protocells can become larger the selection for the replicase is easier.

C. Replicase R1α

In this model the rate of enzymatic replication depends on the amount of A in the protocell and is given by $1 + i\alpha$ where i is the number of enzymes A in the protocell. The rate of duplications $M_{i,j}, L_{i,j}$ are given by

$$\begin{aligned} L_{i,j} &= i(1+\alpha i)q \\ M_{i,j} &= i(1+\alpha i)(1-q) + j(1+i\alpha) \end{aligned} \tag{20}$$

By using the relation Eq. (12) valid at the error threshold, we get that

$$\sum_{i\geq 1,j} i x_{i,j} = q_c \sum_{i\geq 1,j} i(1+\alpha i)x_{i,j} \tag{21}$$

Therefore we have

$$q_c = \frac{\sum_{ij} i x_{i,j}}{\sum_{ij} i x_{i,j} + \alpha \sum_{ij} i^2 x_{i,j}} \tag{22}$$

satisfying the following conditions

$$\frac{1}{1+(m-1)\alpha} \leq q_c \leq \frac{1}{1+\alpha}. \tag{23}$$

Therefore for large values of α the error threshold goes to zero.

We have numerically calculated the error threshold for this model up to $m = 10$ considering both types of protocell division.The results show, as the theory and Eq. (23) predict, that $q_c \leq 1/(1+\alpha)$. Moreover, this bound is tight for large values of α. Finally, the simulation results conducted for the case in which the protocells of maximal size divide into two or into many, show that, for every value of α the error threshold is a decreasing function of m. Therefore if the protocells can become larger the selection for the replicase is easier.

D. Replicase R2α

In this model the rate of enzymatic replication depends on the amount of A in the protocell. If i sequences of type A are present in the protocell, the A sequences replicate at rate $1+\alpha(i-1)$ and the B sequences replicate at rate $1+\alpha i$. In this case the rates of duplication M_{ij}, L_{ij} are given by

$$\begin{array}{lcl} L_{i,j} &=& i[1+\alpha(i-1)]q \qquad\qquad \text{for } \; i\geq 2 \\ M_{i,j} &=& (1+\alpha i)j + i[1+\alpha(i-1)](1-q) \end{array}$$

By using the relation Eq. (12) valid at the error threshold, we get that

$$\sum_{i\geq 1,j} i x_{i,j} = q_c \sum_{i\geq 1,j} i[1+\alpha(i-1)]x_{i,j} \tag{24}$$

Therefore we have

$$q_c = \frac{\sum_{i,j} i x_{i,j}}{\sum_{i,j} i[1+\alpha(i-1)]x_{i,j}} \tag{25}$$

satisfying the condition

$$q_c > \frac{1}{1+(m-2)\alpha}. \tag{26}$$

Moreover by solving the Eqs. (1) in the large α limit we can show that in this limit

$$\begin{array}{ll} x_{1,0} & = d_{1,0}/2 = \mathcal{O}(1) \\ x_{i,j} & = \mathcal{O}\left(\frac{1}{\alpha}\right) \qquad\quad \text{for } \; i\geq 1 \& (i,j)\neq(1,0) \end{array}$$

Therefore, taking into account this scaling and Eq. (17) we can show that

$$q_c(\alpha) \to q_c(\infty) > 0 \;\; \text{as} \;\; \alpha\to\infty \tag{27}$$

We have numerically calculated the error threshold for this model up to $m = 10$ considering both types of protocell division (see figure S2). The simulation results, conducted for the case in which the protocells of maximal size divide into two, show a crossing of the error threshold curves $q_c = q(\alpha)$ calculated for different values of the maximal size of the protocell m, suggesting that, for a value of $\alpha = 100$, there is a optimum size of the protocell. We have conducted the numerical calculations up to values $\alpha = 10,000$. These results suggest that in the large α limit, $\alpha \gg 1$, the error threshold is a decreasing function of the maximal size of the protocell m.

The simulation results conducted for the case in which the protocells of maximal size divide into many, show that, for every value of α the error threshold is a decreasing function of m. Therefore if the protocells can become larger the selection for the replicase is easier.

TABLE S1 : **Maximal length of the selected replicase $L_{\max}$**

Division into two	m	$L_{max}(u=0.17)$	$L_{max}(u=0.0088)$
$R1$	any m	24	521
$R2$	3	4	89
$R2$	4	5	117
$R2$	5	6	130
$R2$	8	6	141
$R2$	10	6	138
$R1\alpha$	3	28	600
$R1\alpha$	4	28	600
$R1\alpha$	5	28	600
$R1\alpha$	8	28	600
$R1\alpha$	10	28	600
$R2\alpha$	3	4	89
$R2\alpha$	4	5	112
$R2\alpha$	5	5	121
$R2\alpha$	8	5	123
$R2\alpha$	10	5	115

Division into many	m	$L_{max}(u=0.17)$	$L_{max}(u=0.0088)$
$R1$	any m	24	521
$R2$	3	2	54
$R2$	4	3	74
$R2$	5	4	86
$R2$	8	5	106
$R2$	10	5	113
$R1\alpha$	3	28	599
$R1\alpha$	4	28	600
$R1\alpha$	5	28	600
$R1\alpha$	8	28	600
$R1\alpha$	10	28	600
$R2\alpha$	3	2	54
$R2\alpha$	4	3	73
$R2\alpha$	5	3	83
$R2\alpha$	8	4	100
$R2\alpha$	10	5	107

Maximal length of the selected replicase $L_{\max}$ calculated by imposing $(1-u)^{L_{max}} = q_c$ for the different models under consideration with $a = 200$ or $\alpha = 200$. The parameter m is the size of the largest protocell.

Appendix A: The fitness of the solution with no A molecules , i.e. $\vec{y}^A = \vec{0}$

When there are no molecules of type A in the protocells, i.e. $\vec{y}^A = \vec{0}$, the eigenvector problem Eqs.(8) becomes

$$C_{BB}\vec{y}^B = \lambda \vec{y}^B \tag{A1}$$

and λ can be obtained from equation

$$|C_{BB} - \lambda I| = 0. \tag{A2}$$

The fitness ϕ is equal to the maximal eigenvalue of the eigenvalue problem in Eq. $(A1)$, i.e. $\phi = \lambda_{max}$ In the case in which we consider the splitting into two daughter protocells the matrix C_{BB} can be explicitly written as

$$C_{BB} = \begin{pmatrix} -1 & & & & & & \frac{(m-1)m}{2^{m-1}-1} \\ 1 & -2 & & & & & \frac{(m-1)\binom{m}{2}}{2^{m-1}-1} \\ & \ddots & \ddots & & & & \vdots \\ & & i-1 & -i & & & \frac{(m-1)\binom{m}{i}}{2^{m-1}-1} \\ & & & \ddots & \ddots & & \vdots \\ & & & & m-3 & -(m-2) & \frac{(m-1)\binom{m}{m-2}}{2^{m-1}-1} \\ & & & & & m-2 & -(m-1)+\frac{(m-1)\binom{m}{m-1}}{2^{m-1}-1} \end{pmatrix}_{(m-1)\times(m-1)} \tag{A3}$$

and therefore $|C_{BB} - \lambda I|$ can be explicitly expressed as

$$|C_{BB} - \lambda I| = (-1)^{m-2}\frac{\Gamma(\lambda+m)}{\Gamma(\lambda+1)}\left[\frac{(m-1)!}{2^{m-1}-1}\sum_{i=1}^{m-1}\frac{\Gamma(\lambda+i)}{\Gamma(\lambda+m)}\cdot\frac{\binom{m}{i}}{(i-1)!} - 1\right] \equiv (-1)^{m-2}\frac{\Gamma(\lambda+m)}{\Gamma(\lambda+1)}\Phi(\lambda). \tag{A4}$$

One should note that

$$\begin{aligned} \Phi(1) &= \frac{(m-1)!}{2^{m-1}-1}\sum_{i=1}^{m-1}\frac{\Gamma(i+1)}{\Gamma(m+1)}\cdot\frac{\binom{m}{i}}{(i-1)!} - 1 \\ &= \frac{1}{m}\sum_{i=1}^{m-1}\frac{i\binom{m}{i}}{2^{m-1}-1} - 1 \\ &= \frac{1}{m}\frac{m2^{m-1}-m}{2^{m-1}-1} - 1 = 0. \end{aligned} \tag{A5}$$

Therefore $\lambda = 1$ is one eigenvalue. Since $\Phi(\lambda)$ is monotonically decreasing function, $\lambda = 1$ is the only real root of Eq.(A2). Similarly, if we consider the splitting of the protocells into many daughter protocells, C_{BB} can be written as

$$C_{BB} = \begin{pmatrix} -1 & & & & & & (m-1)m \\ 1 & -2 & & & & & \\ & \ddots & \ddots & & & & \\ & & i-1 & -i & & & \\ & & & \ddots & \ddots & & \\ & & & & m-3 & -(m-2) & \\ & & & & & m-2 & -(m-1) \end{pmatrix}_{(m-1)\times(m-1)} . \tag{A6}$$

Therefore we can explicitly calculate the following determinant, obtaining

$$|C_{BB} - \lambda I| = (-1)^{m-2}\frac{\Gamma(\lambda+m)}{\Gamma(\lambda+1)}\left[\frac{\Gamma(\lambda+1)}{\Gamma(\lambda+m)}\cdot m! - 1\right] = (-1)^{m-2}\frac{\Gamma(\lambda+m)}{\Gamma(\lambda+1)}\Psi(\lambda). \tag{A7}$$

The function $\Psi(\lambda)$ is a decreasing function of λ, with $\Psi(1) = 0$. Therefore $\lambda = 1$ is the maximal real solution of the system of equations. Finally we conclude that the fitness of a system of protocells in which the molecules A are not present is $\phi = \lambda_{max} = 1$ independently on the splitting mechanism under consideration.

Appendix B: Complete analytic solution of the error threshold for replicase $R2$ with $m = 3$

1. Splitting into two daughter protocells

The quasi-species equations for the frequency of protocells x_{ij} of protocells containing different type of molecules is given by

$$\begin{aligned}
\dot{x}_{1,0} &= -x_{1,0} + (r_{3,0} + \frac{2}{3}r_{2,1} + \frac{1}{3}r_{1,2}) - \phi x_{1,0} \\
\dot{x}_{0,1} &= -x_{0,1} + (\frac{1}{3}r_{2,1} + \frac{2}{3}r_{1,2} + r_{0,3}) - \phi x_{0,1} \\
\dot{x}_{2,0} &= r_{3,0} + \frac{1}{3}r_{2,1} + qx_{1,0} - 2ax_{2,0} - \phi x_{2,0} \\
\dot{x}_{1,1} &= \frac{2}{3}r_{2,1} + \frac{2}{3}r_{1,2} + (1-q)x_{1,0} - (1+a)x_{1,1} - \phi x_{1,1} \\
\dot{x}_{0,2} &= r_{0,3} + \frac{1}{3}r_{1,2} + x_{0,1} - 2x_{0,2} - \phi x_{0,2}
\end{aligned} \quad \text{(B1)}$$

where the rates $r_{3,0}, r_{2,1}, r_{1,2}$ and $r_{0,3}$ are the rate of dissociation of the protocells with three molecules and are given by

$$\begin{aligned}
r_{3,0} &= 2aqx_{2,0} \\
r_{2,1} &= 2a(1-q)x_{2,0} + qx_{1,1} \\
r_{1,2} &= (a+1-q)x_{1,1} \\
r_{0,3} &= 2x_{0,2}.
\end{aligned} \quad \text{(B2)}$$

The fitness ϕ depends on the conservation law that we impose to the system In fact,

- if the total number of protocells $\sum_{ij} x_{ij} = 1$ is conserved then

$$\phi = 2ax_{2,0} + (1+a)x_{1,1} + 2x_{0,2}; \quad \text{(B3)}$$

- if the total number of molecules $\sum_{ij}(i+j)x_{ij} = 1$ is conserved then

$$\phi = x_{1,0} + x_{0,1} + 2ax_{2,0} + (1+a)x_{1,1} + 2x_{0,2}. \quad \text{(B4)}$$

The error threshold of the model is independent on the conservation law that we imposed. To calculate the error threshold $q_c(a)$ we need to take advantage of the structure of the transition matrix shown in Figure .
Therefore let us define the vector $\vec{y}^A$ of the frequency of protocells containing at least one molecule A

$$\vec{y}^A = \begin{pmatrix} x_{1,0} \\ x_{2,0} \\ x_{1,1} \end{pmatrix}$$

and the vector $\vec{y}^B$ of concentration of protocells containing only molecules of type B,

$$\vec{y}^B = \begin{pmatrix} x_{0,1} \\ x_{0,2} \end{pmatrix}.$$

The quasi-species equations given by Eqs $(B1) - (B2)$ read

$$\begin{aligned} \dot{\vec{y}}^A &= C_{AA}\vec{y}^A - \phi\vec{y}^A \\ \dot{\vec{y}}^B &= C_{BA}\vec{y}^A + C_{BB}\vec{y}^B - \phi\vec{y}^B. \end{aligned} \tag{B5}$$

The matrices C_{AA}, C_{BA} and C_{BB} are given by

$$C_{AA} = \begin{pmatrix} -1 & \frac{2}{3}a(2+q) & \frac{1}{3}(1+a+q) \\ q & -\frac{4}{3}(1-q) & \frac{1}{3}q \\ 1-q & \frac{4}{3}a(1-q) & -\frac{1}{3}(1+a) \end{pmatrix}$$

$$C_{BB} = \begin{pmatrix} -1 & 2 \\ 1 & 0 \end{pmatrix}$$

$$C_{BA} = \begin{pmatrix} 0 & \frac{2}{3}a(1-q) & \frac{1}{3}[2(a+1)-q)] \\ 0 & 0 & \frac{1}{3}(a+1-q) \end{pmatrix}.$$

The stationary state is reached when $\phi = \lambda_{max}$ where $\lambda_{max} = 1$ is the maximal eigenvector of the eigenvalue problem

$$\begin{aligned} C_{AA}\vec{y}^A &= \lambda\vec{y}^A \\ C_{BA}\vec{y}^A + C_{BB}\vec{y}^B &= \lambda\vec{y}^B. \end{aligned} \tag{B6}$$

One solution of this system of equation is $\vec{y}^A = \vec{0}$, $\vec{y}^B = (1/2, 1/2)$ associated with the maximal eigenvalue $\lambda = 1$. This solution becomes unstable when the maximal eigenvalue of the eigenvalue problem Eqs. $(B6)$ become grater than one. This occurs at the error threshold $q = q_c(a)$. In order to study when this happens is sufficient to study the spectrum of the eigenvalue problem

$$C_{AA}\vec{y}^A = \lambda\vec{y}^A \tag{B7}$$

and to impose that the maximal eigenvalue of the matrix C_{AA} is equal to one, i.e. $\lambda_{max} = 1$. This happens when

$$\det(C_{AA} - 1) = 0. \tag{B8}$$

This equation is cubic in q and therefore will have three solutions. The only solution in the range $q \in [0, 1]$ provides the required solution $q_c = q_c(a)$ describing the error threshold. The solution to the equation $det(C_{AA} - 1) = 0$ is given by

$$q_c(a) = \frac{2a^2 - 6a - 3}{6a} + \frac{1}{6a}\left[(P_1(a))^2 + 81P_2(a)\right]^{1/6}\left\{\cos(\theta/3) - \sqrt{3}\sin(\theta/3)\right\} \tag{B9}$$

with

$$\begin{aligned} P_1(a) &= 27 + 162a + 2943a^2 + 4968a^3 - 558a^4 - 72a^5 - 8a^6 \\ P_2(a) &= -756a^2 - 5652a^3 - 63621a^4 - 173016a^5 + 366204a^6 + \\ & \quad 197520a^7 + 33484a^8 + 2784a^9 + 128a^{10} \end{aligned}$$

and

$$\tan\theta = \frac{9\sqrt{P_2(a)}}{P_1(a)} \text{ with } \theta \in [0, \pi). \tag{B10}$$

The limits of $q_c(a)$ are $q_c(a) \to 1$ as $a \to 1$ and $q_c(a) \to -2 + \sqrt{6} = 0.449...$ as $a \to \infty$. This error threshold is independent on the constraints that we are imposing (i.e. conservation of the total number of molecules or conservation of the total number of cells).

2. Splitting into many daughter protocells

The quasi-species equations for the frequencies x_{ij} of protocells containing different type of molecules is given by

$$
\begin{aligned}
\dot{x}_{1,0} &= -x_{1,0} + (3r_{3,0} + 2r_{2,1} + 1r_{1,2}) - \phi x_{1,0} \\
\dot{x}_{0,1} &= -x_{0,1} + (r_{2,1} + 2r_{1,2} + 3r_{0,3}) - \phi x_{0,1} \\
\dot{x}_{2,0} &= qx_{1,0} - 2ax_{2,0} - \phi x_{2,0} \\
\dot{x}_{1,1} &= (1-q)x_{1,0} - (1+a)x_{1,1} - \phi x_{1,1} \\
\dot{x}_{0,2} &= x_{0,1} - 2x_{0,2} - \phi x_{0,2}
\end{aligned} \tag{B11}
$$

where the rates $r_{3,0}, r_{2,1}, r_{1,2}$ and $r_{0,3}$ are the rate of dissociation of the protocell with three molecules and are given by

$$
\begin{aligned}
r_{3,0} &= 2aqx_{2,0} \\
r_{2,1} &= 2a(1-q)x_{2,0} + qx_{1,1} \\
r_{1,2} &= (a+1-q)x_{1,1} \\
r_{0,3} &= 2x_{0,2}.
\end{aligned} \tag{B12}
$$

The parameter ϕ depends on the conservation law that we impose to the system.
In fact,

- if the total number of cells $\sum_{ij} x_{ij} = 1$ is conserved then

$$\phi = 4ax_{2,0} + 2(1+a)x_{1,1} + 4x_{0,2}; \tag{B13}$$

- if the total number of molecules $\sum_{ij}(i+j)x_{ij} = 1$ is conserved then

$$\phi = x_{1,0} + x_{0,1} + 2ax_{2,0} + (1+a)x_{1,1} + 2x_{0,2}. \tag{B14}$$

Proceeding as in previous section we get that the error threshold is given by

$$q_c(a) = \frac{-7a - 2a^2 + \sqrt{-12 - 4a + 121a^2 + 100a^3 + 20a^4}}{2(-1 + 2a + 2a^2)}. \tag{B15}$$

The limit for large values of a, i.e. $a \to \infty$ is given by $q_c(a) \to \frac{1}{2}(-1 + \sqrt{5})$.
